# Sewage Sludge Management at District Level: Reduction and Nutrients Recovery via Hydrothermal Carbonization

**DOI:** 10.1007/s12649-022-01943-2

**Published:** 2022-10-05

**Authors:** D. Scrinzi, R. Ferrentino, E. Baù, L. Fiori, G. Andreottola

**Affiliations:** Department of Civil, Environmental and Mechanical Engineering, University of Trento, via Mesiano 77, 38123 Trento, Italy

**Keywords:** HTC, Sludge reduction, Nutrients recovery, Waste management, Economic assessment

## Abstract

In this study, two scenarios of a municipal wastewater treatment plant (WWTP) are presented, which include the integration of the hydrothermal carbonization (HTC) process into the sludge line as a post-treatment of the anaerobic digestion (AD) process. The objective of the simulation is to investigate the performances of AD + HTC treatment to reduce sludge production and improve nutrient and energy recovery. For this purpose, the scheme of an under-construction WWTP was considered, named Trento 3 (Trento, Italy) and with a treatment capacity of 300,000 PE. In the first scenario, the HTC process was fed with thickened sludge from the Trento 3 WWTP, while in the second scenario, dewatered sludge from other local WWTPs was also used as feedstock for the HTC process. Both scenarios allowed to obtain a considerable sludge reduction ranging from 70 to 75% with a notably increase in the biogas production up to 47%, due to the recycling of HTC liquor (HTCL) to the anaerobic digester. Considering nutrients recovery, all the phosphorus and nitrogen present in the HTCL could be used for struvite precipitation with an average yearly gain of 1 million euros. Moreover, the introduction of HTC in the Trento 3 WWTP could allow a reduction in the sludge management costs of up to 2 M€/year.

**Graphical Abstract:**

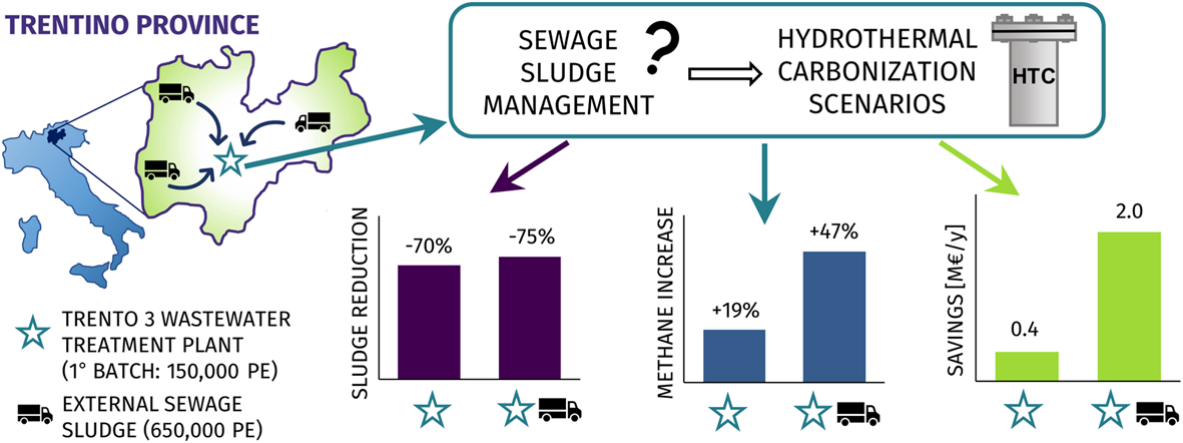

## Statement of Novelty

Nowadays, industrial-scale HTC plants start to be globally available, making the spreading of this technology possible at a large scale, but few studies confirmed the effective benefits for a better waste management. This paper aims to preliminarily assess the integration of HTC at a regional level (province of Trento, Italy), analyzing the historical data and the design parameters of local WWTPs, both from the mass balance point of view and the management costs, referring to some recent literature data on HTC performances. This study can serve as a basis for local administrations, but also policy makers, stakeholders, and industries for future development and actual implementation of the HTC technology in WWTPs. Moreover, it proposes a framework for any new local assessment about HTC applied to sewage sludge management and valorization.

## Introduction

The municipal wastewater treatment sector is estimated to globally produce sludge with a recorded yearly rate of 45 million dry tons [1]. The biggest producers are the United States, China, and the European Union [2]. In 2018, Italy produced more than 3 million tons of raw sewage sludge (i.e., on a wet basis) [3]. Moreover, data in the latest report of the Italian Institute for the Environmental Protection and Research (ISPRA) [3] show a rather constant increase in sewage sludge production in the last four years considered (2015–2018). The processing of excess sludge could represent 25–65% of the total operational costs of a wastewater treatment plant (WWTP) [4]. So far, the sewage sludge produced in the  European Economic Area Member Countries (EEA-32) has four main destinations: agriculture 27%; incineration 25%; compost and other applications 21%; landfill 9%; and other uses for the remaining part (18%) [5]. In Italy, 23% of sewage sludge is sent to landfills while only 8% is sent to incinerators [3]. In this context, the increasing management costs and environmental problems make urgent the application of innovative solutions for the development of technologies for the valorization, reduction and safe disposal of sewage sludge.

Among all possible strategies, hydrothermal carbonization (HTC) of municipal sewage sludge has been recently recognized as a promising technology for efficient waste volume reduction and recovery of bioenergy and nutrients [6, 7], such as phosphorus and nitrogen. HTC is a thermochemical process in which moist biomass is carbonized at a temperature between 180 and 250 °C and a reaction time of 0.5–8 h at saturated vapor pressure, leaving the water in the liquid phase. The three main products of the HTC reactions are the HTC gas, which consists mainly of CO_2_, the HTC liquor (HTCL), a liquid phase (or process water) in which the organic compounds are dissolved due to a series of chemical reactions (e.g., hydrolysis), and a solid phase called hydrochar [8]. The feasibility of the HTC process as a post-treatment of the anaerobic digestion (AD) process to reduce the amount of sludge to be disposed of and to increase the biogas production has already been demonstrated in laboratory tests [9], thus making the coupling of AD + HTC an attractive option [6, 10–12].

As shown in several literature studies, a greater reduction in sludge volume could be achieved with increasing process severity of the HTC process, i.e. increasing temperature and residence time. Recently, Liu et al. [6], in reviewing the HTC of municipal sewage sludge literature, reported that research works showed a mean HTC solid yield, calculated as (mass of hydrochar/mass of feedstock) × 100, dry basis (db), of 60.2% (of which 22.6% was in the 10th percentile, 84.9% in the 90th percentile), highlighting that there was considerable scatter in the data due to the variable severity of the HTC process applied. For example, Berge et al. [13], who used sewage sludge digestate as feedstock for an HTC process at 250 °C for 20 h, obtained a very low solid yield of 47.1%. Kim et al. [14], who investigated HTC treatment to convert sewage sludge digestate into clean solid fuels, reported very different values of solid yield at temperatures between 180 and 280 °C. The results showed reveal that the solid yield at the lowest temperature (i.e. 180 °C) was 93.9%; when the temperature was increased to 220 °C, the solid yield decreased to 88.7%. Further increase in temperature to 250 °C and 280 °C resulted in a decrease in solid yield, which was 83.4% and 80.4%, respectively. Thus, this study confirmed that an increase in process severity, in this case temperature, has a significant effect on the solid yield. These results agree relatively well with those of Aragón-Briceño et al. [15], who studied the effect of temperatures on solid yield of hydrochar from digestate HTC and found that the hydrochar yields were 73.4% at 220 °C and 68.8% at 250 °C.

In addition to the sewage sludge volume reduction, a significant gain is obtained by increasing biogas and biomethane production when the HTCL is fed into an AD plant. As for the sewage sludge reduction, biogas and biomethane yields are also affected by HTC reaction severity: a decrease in biogas production is observed with increasing HTC reaction severity [16]. Literature studies reported that feeding AD with HTCL increased biomethane production between 29 and 60% compared to the conventional AD process [9, 15, 17].

No less relevant are the possible application of HTC in terms of resource recovery, in the form of nitrogen and phosphorus [18], and the possible formation of a recycled fertilizer based on sewage sludge (digestate) [19–21].

From an economic point of view, few studies have evaluated the costs from a regional standpoint, even if some HTC plants are already operating on a large scale [22–25].

Among all Italian regions, Trentino-Alto Adige/Südtirol is the one with the highest specific production (about 121 kg/inhabitant and 130,000 ton/year in 2017) due to its high touristic vocation and high environmental standards. This study aims to collect updated data on the amount of sewage sludges produced and their management and disposal costs (before the spread of the SARS-CoV-2 pandemic and the medium term consequences in this sector). Moreover, these data are used to study the possible implementation of the HTC process in the Trento 3 WWTP, a plant under-construction (expected to be completed by the end of 2022) with a treatment capacity of 300,000 population equivalent (PE), the highest in the province of Trento. Two scenarios were analyzed; the first one considers only the internally produced sludge, while the second one also includes the contribution of sludges discharged from other smaller local WWTPs, which now transfer the sludge to WWTP outside the province. Finally, an assessment of the potential for recovery of nutrients in the form of nitrogen and phosphorus from HTC products through struvite (NH_4_MgPO_4_·6H_2_O) precipitation is presented.

## Sewage Sludge Management in Trentino Province (Italy): State of the Art

Among all sewage sludges produced in the Trentino-Alto Adige/Südtirol region, the Autonomous Province of Trento (PAT) produces about 50,000 ton/year with an average dry content of 20%. This quantity corresponds to the total production of 73 municipal WWTPs with a total treatment capacity of 1,350,000 PE. From 2014 to 2019, the production of sewage sludge increases from 45,000 ton/year in 2014, to 50,500 ton/year in 2019. In order to reduce the volume of sludge to be disposed of, strategies to increase the dry matter content in the dewatered sludge were adopted during these years. However, these solutions only partially contributed to solving the sludge problem, as only a small 1% increase in dry matter content was achieved, resulting in a dewatered sludge with a dry solid content of 20%.

**Fig. 1:**
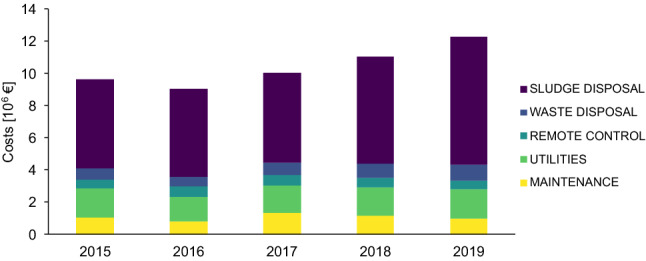
Overall management of WWTP costs in the province of Trento, Italy

Figure 1 shows the costs item for the management of all the WWTPs present in the PAT. This analysis does not include the costs of management established by the procurement contract between PAT and the company that manages the WWTPs, nor the additional costs of ensuring the normal operation of the plants.

Utilities and sludge disposal are the two major cost items in the overall management of a WWTP. The item ‘utilities’ includes energy consumption, in the form of electrical and thermal energy, and water consumption. The cost of this item varied from 19% in 2014 and 15% in 2019 of the total cost, with an average value of 17% in the period considered. In parallel with this reduction in relative costs, there has also been a slight decrease in absolute utility costs, due to the economic agreements reached over the years between PAT and renewable energy producers, which have contributed to a reduction in costs associated with energy consumption. The item ‘sludge disposal’ includes the costs of transporting and disposing of dewatered and dried sludges, which ranges from 58% (2014), to 65% (2019), of total management costs. The data presented in Fig. 1 show that the costs for other items have remained quite stable over the years, while the sludge management costs have increased sharply due to an increase in the average disposal price (including transportation costs) from 110 €/tons of dewatered sludge in 2014 to 150 €/tons in 2019.

**Fig. 2:**
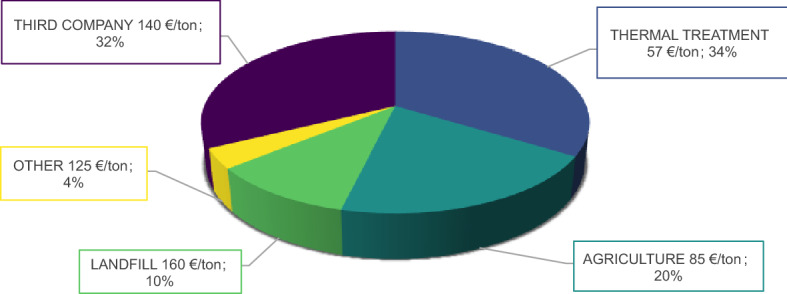
Disposal solution for dewatered sewage sludge in 2019

Figure 2 shows the main solutions and the related average costs for the disposal of dewatered sewage sludge of the PAT in 2019, including thermal treatment, use in agriculture, landfilling and third company take-back. 34% of the total amount of dewatered sewage sludge was sent to thermal treatment, with an average cost of 57 €/ton, while 20% was collected by a private company and used to produce fertilizers, with an average cost of 85 €/ton. However, the highest disposal cost (160 €/ton) are incurred when 10% of the produced sewage sludge is landfilled. In addition, quite high costs (140 €/ton) were paid to third companies that act as intermediaries between the disposal service and other companies that disposed of sewage sludge through composting, direct agricultural application and other processes.

For these reasons, and considering the expected increasing trend in the coming future, an average disposal price of 150 €/ton treated sewage sludge was assumed in the following simulations, excluding the costs due to transport.

In the frame of this regional situation, the PAT is implementing a series of strategies to achieve a gradual reduction in sewage sludge production by (i) reducing the number of small-medium WWTPs, where the sludge treatment line is often not present, and (ii) building a new plant, called Trento 3, with the capability of treating a large amount of feedstock: namely, wastewater for 150,000 PE and sewage sludge for 300,000 PE.

## Implementation of HTC to Reduce Sludge Production

Nowadays, many industries propose commercial HTC systems for the management of wet waste streams [18]. In this section, two simulation scenarios for the application of the HTC process in the Trento 3 WWTP are described. The plant is designed to treat an influent of 96,000 m^3^/d, corresponding to 300,000 PE, mainly coming from the city of Trento and the surrounding municipalities. The water line of the Trento 3 WWTP includes three parallel lines with grit trap and grease separation, 12 lines with primary clarification and chemical phosphorus removal, 4 lines with biological reactors alternating oxic/anoxic phases for the implementation of nitrification and denitrification processes, 24 lines with secondary clarifiers with sludge recirculation and purge followed by tertiary treatments such as flocculation/dephosphorization, filtration, energy recovery and disinfection. The sludge line includes static (2 lines) and dynamic (4 lines) pre-thickening units, ultrasonic treatment, an AD treatment with a post thickening system and, finally, a mechanical dewatering unit. The entire plant includes the biogas treatment and recovery with a gasometer and a cogeneration unit.

However, it is assumed that the WWTP will be divided (and built) in two successive batches, each with a capacity of 150,000 PE each. The main design parameters of this study are related to the first batch and are shown in Table 1.

**Table 1:** Design parameters of the first batch of Trento 3 WWTP

Parameter	Unit	Value
Population equivalent	PE	150,000
Average daily flow	m^3^/day	48,000
Biochemical oxygen demand (BOD_5_)	mg BOD_5_/L	187.5
Total suspended solids (TSS)	mg TSS/L	281.3
Total phosphorus (TP)	mg TP/L	6.25
Total nitrogen (TN)	mg TN/L	37.5

**Fig. 3:**
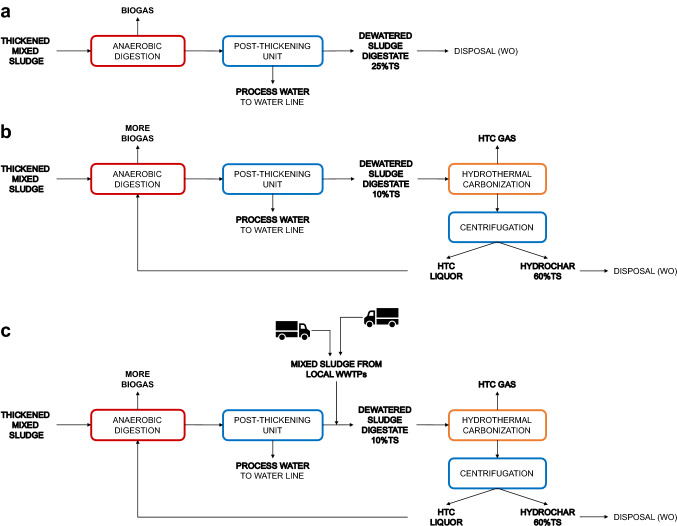
Scenarios proposed in this study. **a** Reference scenario of Trento 3 WWTP, **b** with the implementation of an HTC process after AD with the internal sludge production of Trento 3 WWTP, and **c** with the addition of external dewatered sewage sludges [Adapted from [11]]

Figure 3a shows the reference scenario of the Trento 3 WWTP for the treatment of dewatered digested sludge. Two possible alternative scenarios were considered for the simulation. The first scenario (Fig. 3b) foresees an HTC plant in the sludge line after the AD process to treat only the amount of centrifuged sludge produced internally at the Trento 3 WWTP.

In the second scenario (Fig. 3c), the Trento 3 WWTP becomes a centralized sludge treatment plant, treating sludge from other WWTPs in the surrounding areas; this is a desirable alternative to sludge disposal by companies outside the region, since it involves higher management costs, but also avoidable transportation costs. In all the scenarios considered, for the sake of comparability, it is assumed that the sludge and hydrochar remaining at the end of the process are transferred to an existing local wet oxidation (WO) plant located in Rovereto, Italy, 16 km from the Trento 3 WWTP, which serves as the final destination for the post-treatment of sludge and hydrochar, as described in a previous study [26].

Both scenarios were carried out considering an HTC temperature of 190 °C and a reaction time of 1 h. Under these HTC operating conditions and treating digested sludge with a total solids (TS) concentration of 10%, previous literature studies on the analyzed feedstock have shown the conversion of the solid content into HTC products with a solid yield of 73%, a liquid yield of 26% and a gas yield of 1% [9].

## Results and Discussion

### Reference Scenario

Before applying HTC, some preliminary assessments of the reference scenarios were carried out (Table 2).

**Table 2:** Reference production of centrifuged digested sludge without HTC treatment from plant project

Parameter	Unit	Trento 3	Other local WWTPs
Population equivalent	P.E	150,000	650,000
Reference dry sludge without HTC	ton TS/year	3100^a^	4500^b^
TS	%	25^c^	18.5^b^
Sludge production without HTC	ton/year	12,300	24,500
Methane production without HTC	m^3^/day	2400^a^	–
Initial costs for transportation to WO^e^	€/year	72,700^c^	480,800^c^
Maximum theoretical TP mass in biological sludge	ton TP/year	175^a^	125^d^
Maximum theoretical TN mass in biological sludge	ton TN/year	1050^a^	315^d^

^a^From project design of Trento 3 WWTP

^b^From historical data (2017–2019)

^c^Chosen for this study

^d^From historical data (2019)

^e^WO: wet oxidation plant

According to the design project, the Trento 3 WWTP has a total dry sludge production of digested sewage sludge of about 3100 ton TS/year from the design project, which corresponds to 12,300 ton/year of wet sludge at 25%TS. The plant will also produce about 2400 m^3^ CH_4_/day via AD. The reference production for scenario 2 should also include the external contribution of the selected WWTPs, which refers to about additional 650,000 PE; historical data referred to the years 2017–2019 shows an average of 4500 ton TS/year, corresponding to 24,500 ton/year at 18.5% TS of external sludges. From an economic point of view, local WWTPs which send their sludges out of the province were considered, and the cost of diverting their sludges (production data referred to 2018) to the WO plant was calculated taking into account the average transport cost of 0.371 €/(ton km) of the local contracts. This resulted in annual transport costs of 72,700 € and 480,800 € for the sludges produced in Trento 3 and for the sludge from the other local WWTPs, respectively.

Moreover, according to the design parameters of the Trento 3 WWTP in full operation (300,000 PE; influents with 600 kg TP/day and 3,600 kg N-TKN/day; 80% removal in both wastewater TP and TN), the internally produced sludge will theoretically contain an amount of about 175 and 1050 ton/year in TP and TN, respectively, before AD (scenario 1, Fig. 1b).

### HTC Process Design

According to the design parameters of the Trento 3 WWTP and the new parameters set for the HTC implementation (feedstock with 10% TS), Table 3 shows the calculations for the inlet flow of sludge after AD and dynamic thickening into the HTC reactor, according to the project design of Trento 3.

**Table 3:** Input parameters of centrifuged digested sludge for HTC process design

Parameter	Unit	SCENARIO 1	SCENARIO 2		
			Trento 3	+ other WWTPs	Total
Solid load	kg TS/day	8560	8560	12,400	20,960
TS	%	10	6	18.5	10
Mass flow	kg/day	85,600	143,000	67,100	210,100
	ton/year	31,200		24,500	

The HTC reactor volume was calculated based on the hourly volumetric flow rate of the feedstock and a filling rate of 75%, resulting in less than 5 m^3^ and more than 11 m^3^ for scenarios 1 and 2, respectively. A density of 1033.5 kg/m^3^ was used for the sludge before and after the HTC (around 10% TS), while the density of pure water (1000 kg/m^3^) was assumed for the volume flow of HTCL. Interestingly, from the calculation of the density of hydrochar with 60% TS after filtration as the difference between the initial HTC slurry volume and the HTCL volume flows, the value resulted in 1360–1370 kg/m^3^, but this value should be confirmed by the industrial practice.

The initial dry matter embedded in the feedstock partially solubilized as reflected in the liquid yield value, and partially gasified, so that the dry matter in the two scenarios was reduced by a total of 800 and 2000 ton TS/year, respectively. To simplify the model, it was also assumed that the HTC slurry was dewatered through a filter press or equivalent process in order to obtain a 60% TS hydrochar and an HTCL with a negligible amount of total solids (theoretical capture efficiency of TS: 100%). The HTCL outflow streams were 27,400 and 67,200 ton/year for the two scenarios, respectively, to be recirculated back to the anaerobic digesters. Table 4 reports the results for the main parameters of the HTC reactions and liquid recirculation.

**Table 4:** Results of parameters of the HTC reaction, the quantity of the products and the methane increase due to liquid fraction recirculation

Parameter	Unit	Scenario 1	Scenario 2
HTC inlet volume flow (10% TS)	m^3^/h	3.45	8.46
HTC reactor volume (1 h—75% filling)	m^3^	4.6	11.3
HTC gas	ton/year	31	77
Dry hydrochar produced	ton TS/year	2300	5600
Wet hydrochar (60% TS)	ton/year	3800	9300
Dry mass reduction via HTC	ton TS/year	− 800	− 2000
Sludge total mass reduction via HTC	ton/year	− 8500	− 27,500
Digester inflow from HTCL	m^3^/year	27,400	67,200
Methane production from HTCL	m^3^/day	460	1130

The first significant impact of the HTC process is the reduction of sludge mass: starting from an initial amount of 12,300 ton/year at 25% TS of sludge according to the original project, and an eventual addition of about 24,500 ton/year at 18.5% TS of external sludge, the first scenario allows for a reduction in mass of 8571 ton/year, while the second scenario represents a reduction of 27,500 ton/year, corresponding approximately to − 70% and − 75% less sludge to disposed of, respectively, compared to the reference scenario. Both scenarios resulted in a reduction of solids of about − 25%, due to the solid hydrochar yield.

About 75 m^3^/day for scenario 1 and 184 m^3^/day for scenario 2 of HTCL are recycled to the anaerobic digester, which represents an increase in digester flow of + 28% and + 69%, respectively. Considering a value for chemical oxygen demand (COD) in the HTCL equal to 42.5 kg/m^3^ [27] and a methane production yield equal to 0.144 m^3^ CH_4_/kg COD [28], this results in an increase in the methane production of + 19% and + 47%, respectively for the two scenarios.

While, on the one hand, the mass reduction is significant, on the other hand the limits of these solutions are mainly related to the actual possibility to increase the inlet flow into the digester(s), without excessively reducing the retention time, leading to a possible reduction in biomethane production. As the design of the new plant have a large margin to increase the influent, as it is the case of Trento 3, an increase of + 50% can be reasonably applied under real conditions, thus making scenario 1 feasible. Scenario 2, on the other hand, could only be applied if further analyses on continuous flow anaerobic digestion show that it is possible to reduce the hydraulic retention time without affecting biogas production. Some studies already indicate this possibility. As a matter of fact, while methanogenesis of HTCL is generally slower due to the possible presence of some inhibitors, the hydrolysis rate for anaerobic degradation of HTCL is faster [27, 28]. Moreover, the excess of HTCL in scenario 2 could be used within the WWTP as an internal carbon source for the denitrification process [29].

Many studies have addressed the recirculation of process water in the HTC process [30], but this study underlines the need to fill the gap in the literature on the effect of AD of fresh sewage sludge together with the continuously recirculated HTCL of its digestate in real applications. Furthermore, the effects of wet oxidation on HTC products have not been sufficiently studied yet. Only a few works analyzed the effects of wet oxidation process on HTC products, stating the possibility of oxidizing the dissolved organic matter of process water and hydrochars [31–33]. Further studies could confirm the final properties of the oxidized hydrochar with regards to the circular economy perspective.

### Economic Evaluation

This section presents the economic evaluation for both scenarios. The costs for HTC treatment, disposal, transport and methane production were considered. This evaluation makes it possible to compare the costs required for HTC treatment with the revenues resulting from a lower volume of solid material to be disposed of and transported, compared to that of the dewatered sludge, and from the increase in methane production due to HTCL recirculation to AD.

According to Lucian et al. [34], the cost of HTC treatment is 31 € per tons of sewage sludge treated. This amount was calculated taking into account the energy consumed, the management, and maintenance costs of the process and excluding taxes, costs for property and patent license application, expenses and wastewater treatment (since HTCL would be treated inside the plant itself). Another crucial point to highlight is the assumption that, from a waste management perspective, the wet hydrochar produced is considered equal to the feedstock itself, i.e. digested sewage sludge. This might not be true if the local norms and updated legislation will change the European Waste Code (EWC) of the hydrochar and, consequently, different disposal costs would be incurred.

The amount of sewage sludge fed to the HTC reactor is equal to 31,300 and 76,600 tons/year, respectively, in scenarios 1 and 2. Thus, the cost of the HTC treatment for scenario 1 is equal to 969,000 €/year while that for scenario 2 accounts for 2,374,200 €/year.

The cost for disposal was assessed considering the average price for the sludge disposal of 150 €/tons, as reported above. Thus, the cost of disposing of wet hydrochar disposal is 570,600 and 1,398,200 €/year, respectively for scenarios 1 and 2, having to arrange for the disposal of 3800 and 9300 tons/year, respectively. The cost for wet hydrochar disposal was compared, for scenario 1, with the one related to the disposal of the dewatered sludge produced by Trento 3 WWTP and, for scenario 2, with the sum of the sludge produced from Trento 3 WWTP and the surrounding WWTPs. The Trento 3 WWTP produces an amount of 12,250 tons/year of dewatered sludge, accounting for total costs for disposal of 1,837,500 €/year. Therefore, the implementation of the HTC treatment, according to scenario 1, allows a − 69% reduction in solid waste disposal costs. Moreover, the surrounding WWTPs produce an amount of dewatered sludge of 24,500 tons/year which, together with the sludge production of Trento 3, accounts for 5,512,500 €/year for sludge disposal. Comparing this result with that of the HTC configuration according to scenario 2, the costs for sludge disposal can be reduced by − 75%. Both scenarios, 1 and 2 thus allow for a significant gain, as the amount of solids that need to be disposed of after HTC treatment is greatly reduced. If the final disposal of the wet hydrochar is the thermal treatment, lower disposal costs could be achieved by using the dewatered sludge dryer available within the PAT territory, as the average cost of this treatment is 57 €/tons, without considering the fact that the hydrochar would be delivered with a much lower moisture content.

The total amount of wet hydrochar produced could be transported to the wet oxidation treatment plant, which is about 16 km away from the Trento 3 WWTP. Considering a transport cost equal to 0.371 €/tons of sludge per km, to transport the amount of sludge produced in Trento 3 WWTP, without considering HTC treatment, would be 72,700 €/year instead of 22,100 €/year according to scenario 1. Thus, a saving of 50,600 €/year could be achieved. Obviously, the transport cost to the WO treatment plant is even higher if the sludge produced both in Trento 3 and the surrounding WWTPs has to be transported, as in scenario 2. However, in addition to the cost for sludge transportation from Trento 3 WWTP to the WO treatment plant, the transport cost from the local surrounding WWTPs to Trento 3 WWTP has to be considered, as previously explained. The computed transportation costs for scenario 2 accounts for 429,600 €/year, which means a saving of about 51,200 €/year compared to the original reference value of 480,800 €/year (Table 2).

A significant gain could be achieved by increasing the biogas production due to the feeding of HTCL into the AD unit. Assuming a COD concentration of HTCL equal to 41 kg COD/m^3^, a methane production of 0.144 m^3^ CH_4_/ kg COD_added_ [9] and a production of HTCL in scenario 1 equal to 27,400 tons/year, a methane production of 161,900 m^3^/year is obtained. Moreover, assuming an average methane price for industrial consumers equal to 0.30 €/m^3^ (reference year 2019, EU area) [35], the revenue from methane sales for scenario 1 amounts to 48,600 €/year. In scenario 2, where the HTCL that could be fed into the AD accounts for 67,200 tons/year, corresponding to a methane production of 396,700 m^3^/year, a final gain up to 119,000 €/year can be achieved. Table 5 summarizes the results of the economical evaluation for both scenarios 1 and 2.

**Table 5:** Economic evaluations for the two scenarios

Item of expenditure [€/year]	Scenario 1	Scenario 2
Costs for HTC treatment	− 969,000	− 2,374,200
Savings from reduced final hydrochar disposal	1,267,000	4,114,300
Savings from reduced sludge transportation	50,000	51,200
Gains from increased methane production	49,000	119,000
Total gains	397,000	1,910,300

Although the reported results are case-specific, and despite the fact that the sewage sludge will be managed by a public service that can provide ad hoc incentives and investments, they can be compared to the values in the literature.

The total investments vary depending on the construction and commissioning of the plant, utilities and contingencies, and may affect the payback period or even the feasibility of the proposed scenarios. A case study similar to scenario 1 is reported by Lucian et al. [23], who designed the process and the plant to treat 20,000 ton/year of grape marc with 35% TS treated via HTC at 220 °C for 1 h. The total investment required was about 1.8 M€. Another study conducted by Medina–Martos [25] evaluated the techno-economic characteristics and life cycle assessment of HTC at 280 °C for 1 h of 2970 ton/year of secondary sewage sludge followed by co-digestion of 4530 ton/year of primary sewage sludge and HTCL, compared to AD of the mixed sludge. In this case, the calculation also included the construction and management of the AD. The authors found that the total capital cost over a 20-year lifetime was 25.7 M€ for the first option compared to 18.9 M€ for the second option. Based on these values, we can assume that the implementation of the HTC plant alone could theoretically make up the difference of 6.8 M€. According to these studies, the range for capital costs is between 1.8 and 6.8 M€, corresponding to 4.5–17 times the total profit given in Table 5, respectively.

For scenario 2 (76,700 ton/year of sludge treated), there is a similar sized case study by Ciceri et al. [36], where 78,000 tons/year of biowaste (30% TS) can be treated with an initial investment of 27.3 M€, which is 14 times the total profits evaluated in this work. The operating costs for HTC treatment are also comparable, as they are reported to be around 3 M€/ear.

As suggested by Saba et al. [37], the size of the plant should be carefully considered to find a trade-off between lower costs due to economies of scale and higher expenses due to increased capital and manufacturing costs of the plants. According to their sensitivity analyses, the team also remarked the fact that the hydrochar solid yield has a stronger effect on the change in production costs than the effect of scaling, thus suggesting the need for double checking the adherence of the yields at large scale as a crucial parameter.

### Nutrients Recovery Assessment

This section describes a preliminary assessment of nutrients recovery. The HTC process enables and facilitates the nutrients recovery, such as phosphorus and ammonium, from sewage sludge. The distribution of nutrients among the initial sludge, HTCL, and dry HTC solid phase was evaluated based on previous studies on a local WWTP [9, 17], and it is reported in Table 6. For simplicity, HTCL is considered only as a liquid, while hydrochar (HC) is solid only in its dry basis (db) form. Ammonium is considered solubilized only in its liquid form.

**Table 6:** Mass balance of the nitrogen and phosphorus forms along the process of 1 ton of sewage sludge digestate, according to previous studies [9, 17]

Parameter	Unit	Sewage sludge digestate	HTC slurry	HTCL-tot	HC (db)
TS	%	10.0	7.3	0	100.0
Mass	kg	1000	988	916	72
Kjeldahl N (N-TKN)	kg N-TKN	7.2	7.2	3.8	3.4
Ammoniacal N (N-NH_4_)	kg N-NH_4_	2.2	2.5	2.5	0.0
Total P (TP)	kg TP	4.4	4.4	0.05	4.4
Orthophosphate (P-ortho)	kg P-ortho	0.88	0.05	0.05	0.004
Ammoniacal N ratio	mol N-NH_4_/mol N-TKN	0.31	0.34	0.64	0.00
Ammonium to TP ratio	mol N-NH_4_/mol TP	1.1	1.2	120	0

The HTC slurry has a moisture content of 92.7% [9]. While TP and N-TKN are considered constant for mass conservation after HTC, which might not always be the case for nitrogen under real conditions due to ammoniacal emissions, the N–NH_4_ content after HTC can be about 34% of the N-TKN content in moles, starting from an initial value of 31% (corresponding to an increase of about + 12% of the N–NH_4_ content due to the mineralization of N by HTC) [17, 18].

**Fig. 4:**
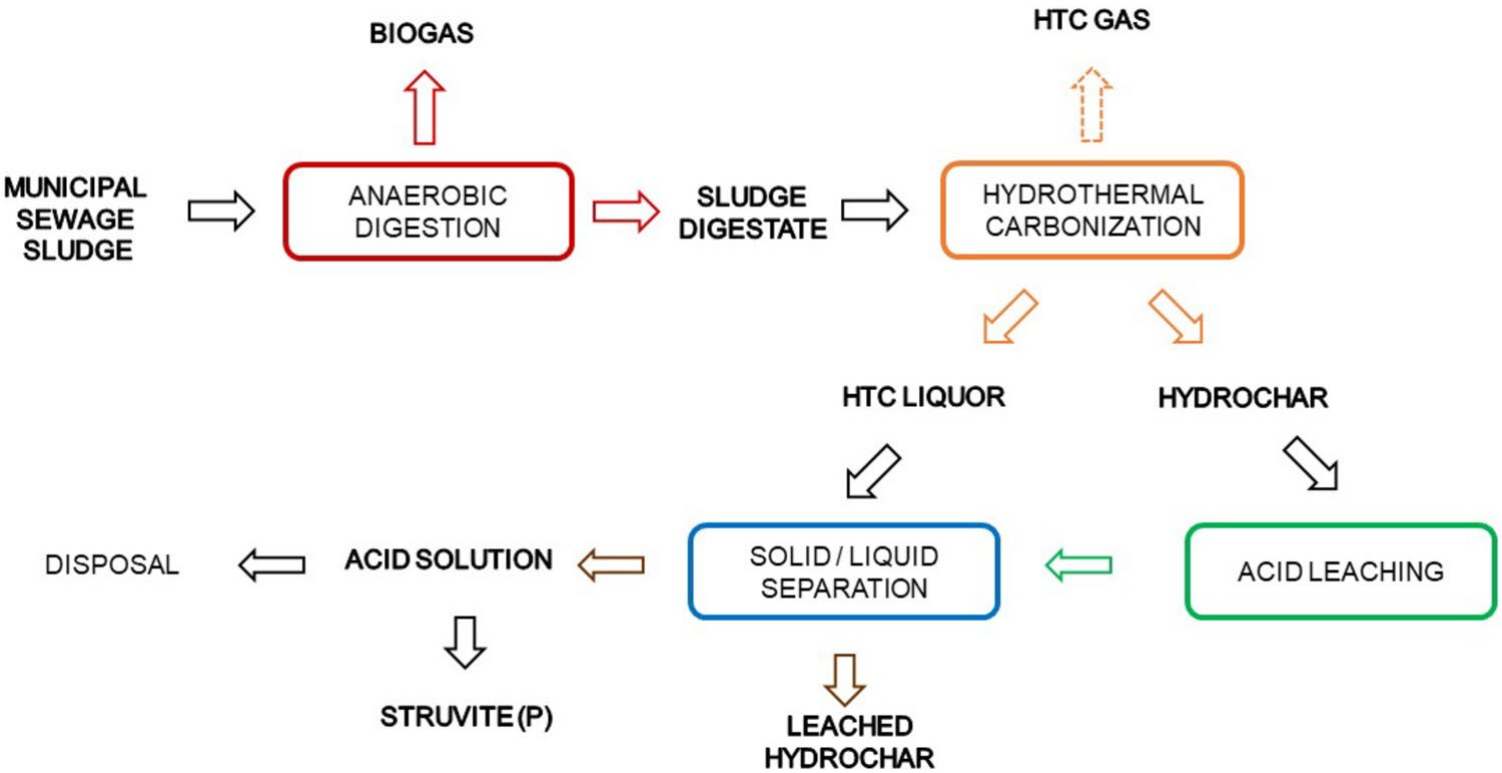
Process flow considered for nutrients recovery via HTC at Trento 3 WWTP

Another crucial factor is the molar ratio between N-NH_4_ and TP, as it gives an indication of the excess of N-NH_4_ over P. As this ratio increases from 1.1 before the HTC process to 1.2, the N-NH_4_ is still sufficient for struvite precipitation and no further nitrogen recovery by ammonium stripping is possible. This puts N recovery from the HTCL in competition with recycling to the anaerobic digester, making this option as an alternative scenario. Therefore, to avoid any ammonium loss and to minimize the amount of acid for phosphorus extraction from the wet hydrochar, the procedure proposed by Becker et al. [38] could be applied (Fig. 4).

The first step after HTC of the sewage sludge digestate (carried out at 190 °C for 1 h) is the separation of HTCL from wet HC, which does not need to reach a high value in TS. Subsequently, acid extraction with citric acid leaches the phosphorus into a soluble form. After acid extraction, the phosphate rich leachate is mixed with the ammonium-rich HTCL and struvite precipitation is promoted by adding a magnesium source, such as MgCl_2_, and the necessary amount of NaOH to reach a pH of 9. It has been reported that the overall process recovers 82.5% of phosphorus in the form of struvite [38].

Table 7 shows the mass balance relevant to nutrient recovery from the amounts of TP and TN, which were considered equal to 125 and 315 ton/year, respectively, using the weighted average historical data of 2019. As phosphorus is the stoichiometrically limiting agent in struvite precipitation, the calculation is based on the recovery of P as struvite, then the total recovered N is calculated backwards to find the recovery efficiency. The average price of struvite in the fertilizer market is €475.5 per ton, according to Aragón-Briceño et al. [22], in accordance to the range 188–763 €/ton struvite found by Molinos-Senante et al. [39].

**Table 7:** Mass balance and recovery efficiency (η_r_) for the potential nutrient recovery as struvite from Trento 3 WWTP, as in scenario 1, and the digested sewage sludge coming from the other local WWTPs, as in scenario 2

		Ton/year	Ton/year recovered	η_r_	Ton/year lost	Ton struvite/year	€/year
Trento 3	TP	138^a^	113	82.5%	24	899	427,500
	TN	225^a^	51	14.8%	174		
+ Other WWTPs	TP	125^b^	103	82.5%	22	817	388,600
	TN	315^b^	47	22.8%	268		
Total (η_r_ 82.5%)						1716	816,100
Maximum (η_r_ 100%)						2080	989,200

^a^From Table 6

^b^From historical data (2019)

The estimated amount of phosphorus that can be recovered as struvite precipitate by the HTC process is therefore around 113 and 216 ton TP/y for scenarios 1 and 2, respectively, while the nitrogen recovered would be 51 and 47 ton TN/y, respectively. The production of struvite in scenario 1 can be calculated as about 5 kg/100 m^3^ of wastewater entering the plant, far more than the 1 kg/100 m^3^ suggested in other studies [39].

Given the high selling price of struvite, the gains from this fertilizer range from about 430 k€/year from the sludge of the Trento 3 WWTP to about 800 k€/y when external contributions are also taken into account. The maximum theoretical total production of struvite (100% of P recovery as struvite) in the centralized plant would be 2000 ton/year, corresponding to revenues of almost 1 M€/year. With a recovery efficiency of 82.5%, scenario 1 would allow a net revenue of about 352,700 €/year starting from 31,200 ton/year of initial sewage sludge to be treated (Table 3), which means an economic benefit of about 11.3 €/ton of treated sludge. Scenario 2, on the other hand, would treat 55,700 ton/year of mixed sludge (Table 3) with a net income of 816,100 €/year from the sale of struvite, which corresponds to about 14.7 €/ton of treated sludge. These values are consistent with a similar study by Aragón-Briceño et al. [22], which found that the income of economic benefits ranged from 9.1 to 18.1 €/ton of sludge for scenarios with HTC at 160 °C and from 20.2 to 26.2 €/ton for scenarios considering thermal treatment at 250 °C.

It should be noted that the values obtained in this study are far higher than the revenues resulting from the increase in methane production due to HTCL recycling into AD (Table 5), which makes this perspective attractive from an economic point of view. However, these gains should be reduced by the costs of reagents (acids and bases involved in the process), and operating costs resulting from energy consumption, maintenance and management of the additional unit in the Trento 3 WWTP, without also mentioning the capital costs for the design and construction of the related facilities. All these costs are specific to the substrate and the operating conditions, and could be analyzed in depth with further studies on the real substrates. Many studies have analyzed the costs of phosphorus recovery, sometimes highlighting the excessive costs in phosphorus production. Munir et al. [40] evaluated a preliminary analysis that only considered the cost of chemicals and excluded the cost of energy, chemical recovery or initial capital costs. They found that the maximum net income occurred for the case of phosphorus recovery after wet oxidation at 200 °C for 1 h rather than after HTC at 180 °C for 1 h (net income equal to NZ$14.95/100 m^3^ of treated sludge versus NZ$ 3.51/100 m^3^, respectively), suggesting a different scenario that should be considered in further studies.

As regards to the operational costs, they can vary between 1.6 and 8.8 € per kg of P recovered, corresponding to about 200–1000 €/ton of struvite recovered [41, 42], implying that the selling price may be exceed depending on operating conditions. This cost could even be reduced to 0.27 $/kg of P recovered (corresponding to 34 $/ton of struvite recovered) if economical magnesium sources, such as seawater nanofiltration brine, were used [42].

On the other hand, capital investments could be comparable to those reviewed by Molinos–Senante [39], suggesting an investment cost of about 1,4 M€ for the recovery of P from sludge in a 100,000 PE WWTP, which is four times the annual income deriving from scenario 1 with a recovery efficiency of 82.5% (about 350 k€/year).

In terms of legislation, the regulatory framework for the use and trade in salts precipitates as fertilizers is constantly evolving. The European Commission has recently added new elements allowed as sources of precipitates [43], including sewage sludge treated with anaerobic digestion and thermal hydrolysis up to 275 °C (thus, the HTC process is included in this definition). In this context, struvite precipitate produced from sewage sludge digestate after HTC could be used and sold on the European market if the final product will meet the chemical composition requirements expressed by the EU Fertilization Directive 2019/1009 [44]. Nevertheless, some incentives might be necessary to make the final product more competitive than mineral fertilizers [45].

If nutrient recovery is not possible or economically competitive, another alternative could be to use hydrochar as a substitute for fossil fuels, which is already allowed by the Italian Norm UNI 11853:2022 [46]. Other uses are still under investigation and are not yet feasible on a large scale, also due to legal barriers [10], such as the direct use of hydrochar as a soil amender-fertilizer or as an adsorbent material for water remediation [6]. In addition, the alkaline liquid byproduct could be ideally recycled back to the water line if the thresholds for nutrients and pollutants were fulfilled, or properly treated with further costs [6].

## Conclusions

The simulation scenarios presented in this study show the feasibility of applying the HTC technology to the full-scale WWTP of Trento 3, which represents a case study and whose results could be extrapolated to other WWTPs. Two scenarios were analyzed: in scenario 1, an HTC plant would treat only the digested sludge generated in the first batch of the Trento 3 WWTP (150,000 PE), while scenario 2 also considers the collection of local external sludge to increase municipal sludge treatment performance at district level (other 650,000 PE).

The main results are reported as follows.

- A great sludge reduction ranging from 70 to 75% depending on the scenario and an increase in methane production, reaching up to 47% in scenario 2, due to the recycling of HTCL to the anaerobic digester.
- From an economic point of view, the introduction of HTC would allow a reduction in management costs of up to 2 M€/year if Trento 3 became a centralized treatment system for other local WWTPs, so that all the sewage sludge and digestate produced in the province of Trento would be treated only on site. The costs associated with taxes, royalties, ownership and patent applications were not considered in this study.
- From a nutrient recovery standpoint, given the availability of P and N in the feedstock, all of the HTCL should be used as a nitrogen source for struvite precipitation, preventing further use for biomethane production. Selling about 2000 tons of struvite per year as fertilizer could generate a theoretical maximum value of 1 million per year, depending on recovery efficiency. However, the new costs of the additional leaching and precipitation equipment, the reagents, and the disposal of the liquid by-product should be taken into account and properly analyzed to obtain a more consistent assessment of the economic benefits of such a challenging treatment path.

For all these reasons, HTC implementation should be considered among all the possible treatment scenarios when dealing with sewage sludge management at district level. Further studies applied to pilot or large-scale plants will help filling some missing gaps in the literature, like the effect of continuous recirculation of HTCL together with the sewage sludge in AD, or the fate of the final byproducts after nutrients recovery.

## Data Availability

The datasets generated and analyzed during the current study are not publicly available, but are available from the corresponding author on reasonable request.
